# Confirming—and testing—bonds of trust: A mixed methods study exploring community health workers’ experiences during the COVID-19 pandemic in Bangladesh, Haiti and Kenya

**DOI:** 10.1371/journal.pgph.0000595

**Published:** 2022-10-07

**Authors:** Pooja Sripad, Ann Gottert, Timothy Abuya, Alain Casseus, Sharif Hossain, Smisha Agarwal, Charlotte E. Warren

**Affiliations:** 1 Population Council, Washington, DC, United States of America; 2 Population Council, Nairobi, Kenya; 3 Zanmi Lasante, Port-au-Prince, Haiti; 4 Population Council, Dhaka, Bangladesh; 5 Johns Hopkins University, Baltimore, MD, United States of America; Universitas Sebelas Maret Fakultas Kedokteran, INDONESIA

## Abstract

Amidst the COVID-19 pandemic and national responses, trust (one’s belief that a system acts in one’s best interest) is important to consider. In community health systems, trust is embedded in relationships between clients, CHWs, and health system stakeholders. This mixed-methods study explores trust through the evolving COVID-19 crisis in Bangladesh, Haiti, and Kenya, where multi-country community health research was underway. We investigate the extent and ways trust between communities, community health workers (CHWs), and health system actors shift, including its relation to community fear and hostility, through self-reported positive and negative experiences of CHWs and policy/program stakeholders on a phone-based survey with 2,025 CHWs and 72 key informant interviews, including CHWs, in late 2020. On surveys, CHWs reported high levels of community trust (8/10 in Bangladesh and Kenya; 6/10 in Haiti) with over 60% reporting client relief in seeing their CHWs. About one-third of CHWs across countries reported experiencing instances of hostility from community members during the pandemic in the form of refused home-entry, ignored advice, or being shouted at. Multivariate analyses revealed that CHWs reporting more positive and fewer negative experiences is consistently associated with continuing routine work, doing COVID-19-related work, and greater community trust. Qualitative interviews showed that existing pre-pandemic trusting relationships withstood the early phase of COVID-19, mitigating negative community reactions toward CHWs and stigma towards COVID-positive individuals, maintaining routine health services, and sustaining appreciation for CHW-provided prevention information and emotional support. CHW-community and CHW-health system actor trust is strengthened when CHWs are well-resourced; CHW-community trust is strained by public frustration at the pandemic, associated restrictions, and sociopolitical stressors. Our study suggests that with adequate institutional support, bonds of trust can promote resilient community health systems during extended public health crises, through CHWs’ commitment to mitigating misinformation, reducing stigma, maintaining routine service provision, and promoting COVID-19 prevention.

## Introduction

Community health workers (CHWs) are highly trusted within community health systems globally, providing a range of reproductive, maternal, newborn, child,(RMNCH) and primary healthcare (PHC) services including education and health promotion. CHWs–frontline workers with up to six months of initial training who provide care in community settings, particularly for those living in low-income or rural communities with limited access to facility-based care–are intermediaries between community members and the broader health facility structures that house more advanced clinical personnel. As such they are often the first points of contact and facilitate linkages to advanced health care across low- and middle-income countries [1]. In Bangladesh, Haiti, and Kenya, community trust in CHWs is a function of their health care competence and ability to respectfully communicate, and is closely related to experienced interactions [2]. Trust within health systems relationships necessitates a risk calculation by one actor or another, whereby faith is placed that someone with higher knowledge or power (e.g. CHW or facility-based supervisor/manager) will act in the best interest of the other (e.g. client or CHW) [3, 4]. Risk calculation and the nature of interactions experienced between clients, CHWs, and other health system stakeholders can change in the face of a shock like the global COVID-19 pandemic [5]. Such change can give rise to confusion spurred by misinformation and fears of contagion.

During infectious disease outbreaks such as the COVID-19 pandemic, facility-based services become inaccessible to many. In these contexts CHWs–globally and in Bangladesh, Haiti, and Kenya, specifically–continue to serve their integral role as trustworthy sources of health information, prevention and care, often performing their routine duties with added responsibilities (e.g. surveillance) and limited support [6–10]. The COVID-19 pandemic, similar to humanitarian settings such as Haiti, Burkina Faso, Democratic Republic of Congo, and South Sudan, creates conditions that unveil the need to protect all healthcare providers, including CHWs, so that they feel safe and supported in their work, for example through the provision of personal protective equipment (PPE), supportive supervision, and mental health care [11–13]. While healthcare provider perspectives are somewhat studied in humanitarian settings with a focus on technical capacity, communications skills, and resources and guidelines availability [14], there has been less emphasis on understanding the sociocultural dimensions of positive and negative experiences [15]. Even when healthcare provider experiences are captured as in China, Turkey, Iraq, and Lebanon, there is inadequate disaggregation of experiences between worker cadres and less emphasis on CHWs working in intermediary roles and lower levels of a health system hierarchy [16, 17]. How CHWs’ social experiences of care provision affect their relationships with clients and communities, including the protective effect of trust in CHWs during disease outbreaks and pandemics merits investigation.

Several critical questions arise from the current COVID-19 pandemic situation in which CHWs are working as frontline service providers and educators in the context of the highly contagious COVID-19 virus. How does disease-associated stigma play into their ability to sustain work? In the context of COVID-19 in Nigeria, Rwanda, South Africa, and Uganda, where perceived and enacted COVID-19 associated stigma is an emergent issue at the community level, CHWs engaging with contact tracing that are identified as having contact with a positive case may be labeled as harbingers of that stigma [18]. How does this impact the established trust between the CHWs and the communities they serve? Are CHWs able to maintain a health systems accountability to and trust of the community? How do risk calculations embedded in trusting relationships shift or withstand the pandemic’s shock to the health system? A rapid evidence review of CHW performance in pandemic contexts (SARS, MERS, Ebola and Zika) building on the Community Health Performance Measurement Framework [19], suggests that not only is it challenging for CHWs to prioritize varied responsibilities while adopting new ones without clear government guidance and support, but the lack of health system and community support may affect the trusted intermediary relationship they serve [6]. Beyond the framework-identified inputs (logistics, funding), supportive systems, CHW competencies and well-being domains that affect CHW performance in a pandemic, the community-centric outcomes–trust and quality of community interactions are critical to explore. Further investigation is required to situate CHW experience and unpack the trust in and value of community health under these circumstances.

As a part of the Frontline Health project that aims to measure, build evidence, and strengthen community health systems’ performance globally [15], we found high levels of pre-pandemic trust in CHWs in Bangladesh, Haiti and Kenya [2]. In the current study, by finding out about positive and negative experiences of CHWs, we explore how the protective effect of trust withholds throughout the dynamic COVID-19 crisis in each of these settings. We investigated the following research questions:

1. To what extent, and in what ways, does trust between communities and CHWs protect and sustain community health services during COVID-19 pandemic?
2. How do CHWs experience community fear and hostility in the context of COVID-19?
3. How is the quality of relationships between CHWs and health facilities affected by the context of COVID-19?

## Methods and materials

### Ethics statement

This study was approved by the Population Council Institutional Review Board (IRB) (p942) as well as the AMREF Health Africa Ethics and Social Review Committee in Kenya (P836-2020), the Bangladesh Medical Research Council (32228072020), and the Zanmi Lasante IRB in Haiti (ZLIRB 08192020). All participants provided verbal informed consent to participate in the study. Compensation of 500 HTG (6 USD), 200 KES (2 USD), and 200 and 500 BDT (2.5 and 5 USD, for CHWs and supervisors) was provided in Haiti, Kenya, and Bangladesh, respectively, to cover airtime and phone charging costs to ensure participants’ ability to stay on the phone for the duration of the survey or interview.

### Study design

We conducted a mixed-methods concurrent exploratory study to understand the perspectives of CHWs and policy/program stakeholders about CHWs’ positive and negative experiences interacting with communities and health facilities during the COVID-19 pandemic. Surveys conducted with 2,025 CHWs quantitatively explored the frequency of these positive and negative experiences. Key informant interviews (KIIs) with 72 purposively sampled community health stakeholders, including CHWs, policy makers, and program implementers, qualitatively elicited the nature and quality of interactions CHWs sustained with communities and facilities. To assess the protective effect of trust we investigated quantitative measures and qualitative accounts of CHW-experienced relationships and interactions—positive and negative. Triangulating thematic areas across methods allowed for a more nuanced understanding of how trust, among other factors, is associated with positive and negative CHW experiences in during the dynamic COVID-19 context.

### Participants and setting

**Fig 1:**
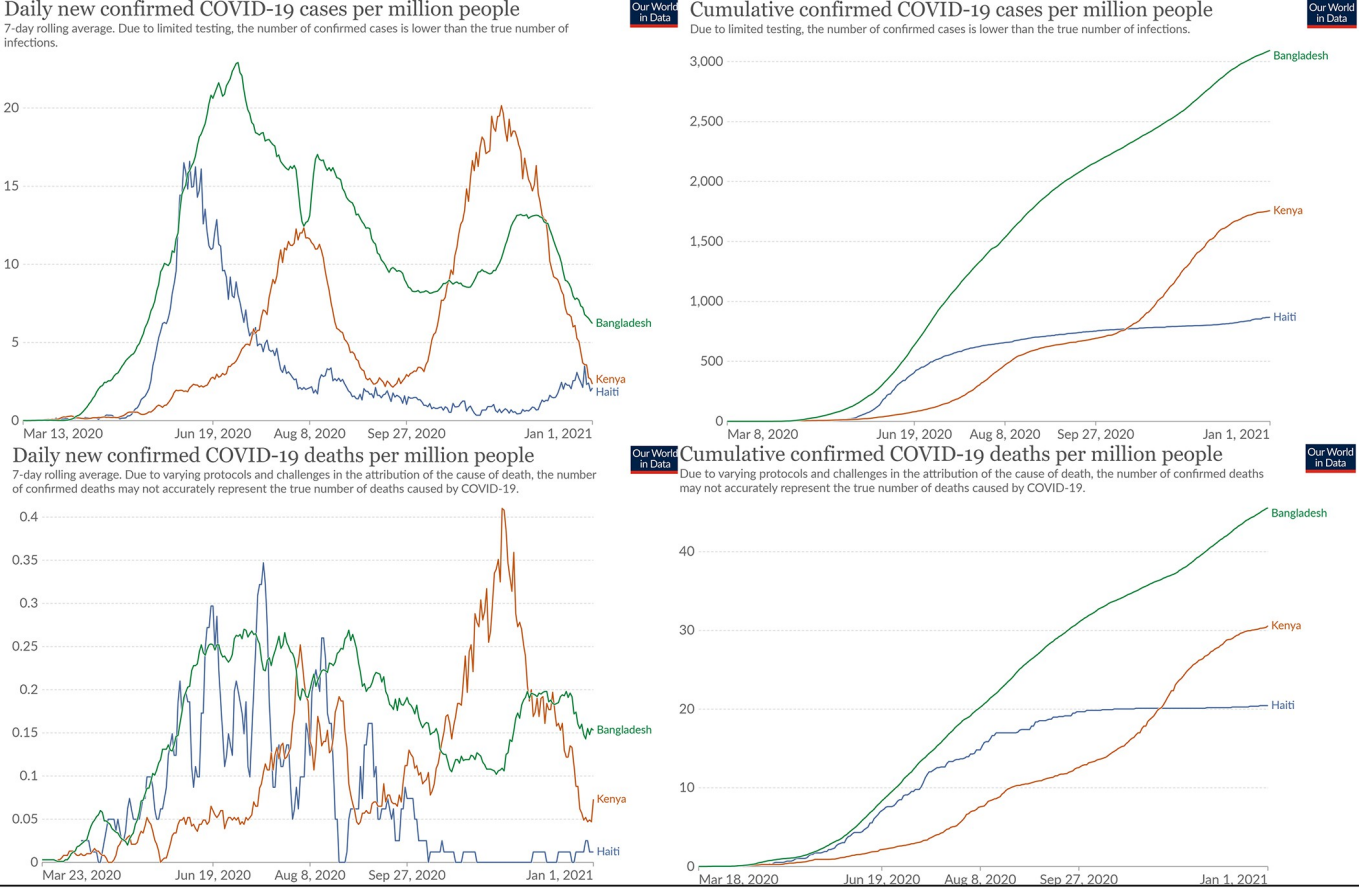
Trends in COVID-19 burden in Bangladesh, Haiti, and Kenya (March–December 2020). Source: Johns Hopkins CCSE COVID-19 Data, accessed from https://ourworldindata.org/coronavirus/.

**Fig 2:**
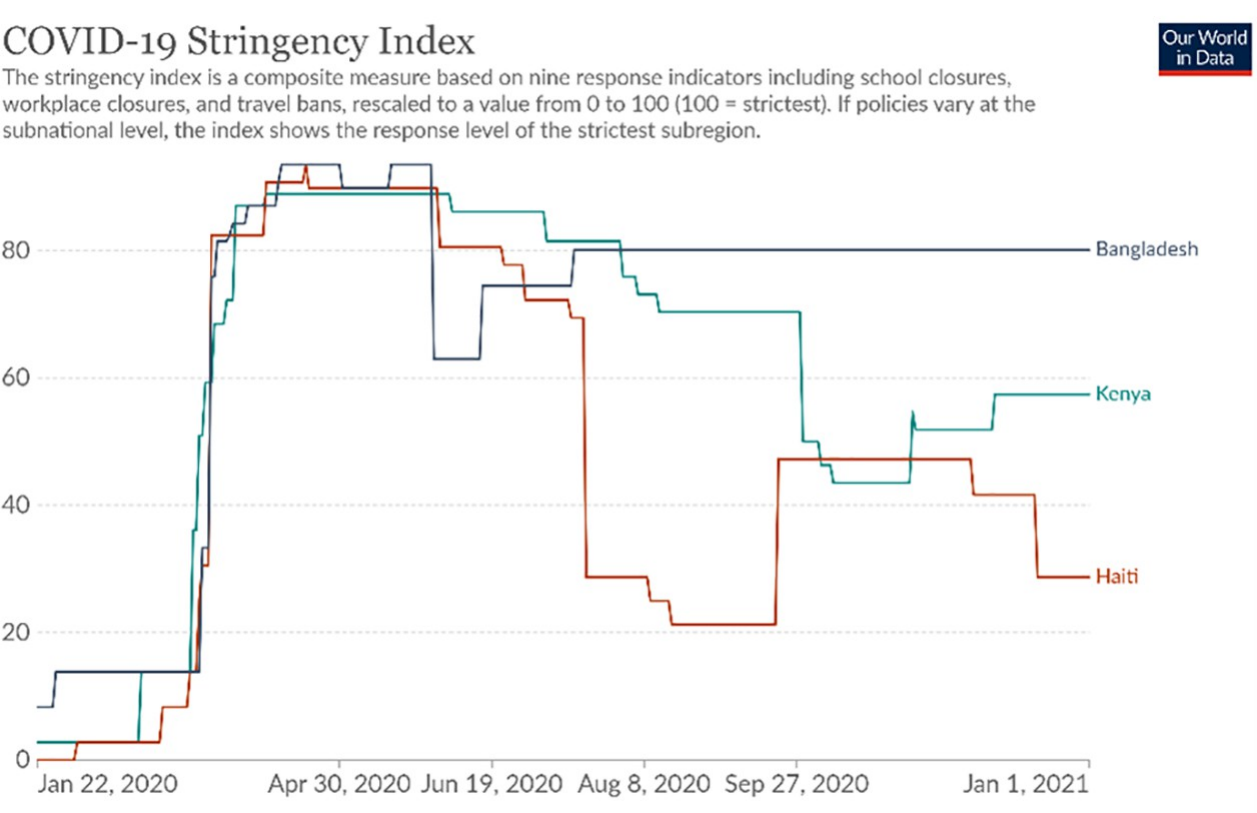
Government restrictions in Bangladesh, Haiti, and Kenya (March–December 2020). Source: Oxford COVID-19 Government Response Tracker, Blavatnik School of Government, University of Oxford, accessed from https://ourworldindata.org/coronavirus/.

We collected cross-sectional phone-based surveys from urban/peri-urban and rural areas in Kenya, Bangladesh, and Haiti between September and December 2020 –thus investigating experiences 6–8 months into the pandemic. Governmental responses to the pandemic in these countries began in March 2020 and included stay-at-home orders, curfews, travel restrictions and school closures. The COVID-19 burden was highest in Bangladesh, moderate in Kenya, and low in Haiti as measured by fluctuating trends in new and cumulative confirmed COVID-19 cases and deaths as shown in Fig 1 [20, 21]. A stringency index (range 1–100) that tracks government responses over the course of pandemic, calculated by aggregating school and workplace closures, stay-at-home orders, and limits on public gatherings and transport, shows that government restrictions across all three countries were high (80–90) between March and July (Fig 2) [20, 22]. After that, restriction patterns are inversely associated with COVID-19 burden, placing Bangladesh’s government response as most, to Kenya as moderately, and Haiti as least stringent (Fig 2) [20, 22]. For example, In Kenya, toward the end of 2020, while isolation of positive cases and large indoor and outdoor gathering restrictions (e.g. places of worship, political rallies) continued, curfews, in-restaurant dining, small gathering, and some travel movement restrictions were lifted [23]. In Haiti, while initial lockdowns affected certain group-engaging community health activities such as CHW-led rallies and vaccination posts, home visits continued throughout this time period; moreover, many restrictions eased by August.

CHWs in all three countries were part of promoting protective measures in communities within the broader COVID-19 response and were provided some training and PPE, through existing supervisory structures [8, 10, 24]. Our study’s quantitative survey participants included CHWs supported by both government and non-governmental organizations (NGOs). Local CHW cadres included ‘Community Health Volunteers’ across 39 out of the country’s 47 counties in Kenya, ‘Family Welfare Assistants’ and ‘Health Assistants’ in four districts in Bangladesh where the Council conducted recent studies, and ‘Agents Santé Communautaire Polyvalent’ from two Departments supported by the Ministry of Health and the NGO Zanmi Lasante in Haiti. CHWs were proportionally representative of rural and urban/peri-urban CHWs in Haiti and Bangladesh, while rural CHWs were oversampled in Kenya. Qualitative KIIs drew on a subset of CHWs surveyed as well as purposively selected community health policy and program stakeholders at national and subnational levels. KII participants included government officials, non-governmental program managers, and community health focal persons and coordinators. The study team selected participants through their networks and on-going research/program activities under the Frontline Health project.

### Data collection

The phone-based surveys and KIIs took 30–45 minutes or an hour, respectively, to complete. CHWs were recruited through a two call process, used an authentication code, and provided informed consent verbally or by SMS. Data were collected by experienced interviewers trained on the study protocol and research ethics, who had at least a bachelor’s degree. Interviewers in each country included women and men between 20–40 years old. If respondents did not pick up the initial call, they were called back up to three times in Bangladesh, Haiti, and Kenya. Across the settings, CHWS mostly called on their own phones and response rates were high (95–98%), as documented through fieldnotes and reported by data managers.

For surveys, sample size estimates of 200 per country were calculated for exploratory studies, using total CHWs population sizes (~65,000 in Kenya, ~6000 in Haiti, ~34,796 in Bangladesh), a 95% confidence interval, 8% margin of error, and a 50% non-response adjustment; final samples were determined by pragmatic decisions at country level, CHW programmatic reach, and a low non-response. In Kenya, our sample included country-wide representation (n = 1,385) and CHWs were randomly sampled based on Ministry of Health rosters for the 39 counties. In Bangladesh (n = 370), proportional sampling was conducted of Family Welfare Assistants and Health Assistants, using our research team’s existing contact information for CHWs working in the four districts. And in Haiti (n = 270), CHWs were randomly sampled from Ministry of Health/Zanmi Lasante listings of CHWs within the two departments. The survey comprised closed- and open-ended questions aimed at assessing the magnitude of CHW experiences as they relate to trust. Survey measures of CHWs’ positive and negative experiences were mainly created by the multi-country-based study team, rather than drawn from validated sources, given the nascency of the COVID pandemic and relevant community health studies.

A total of 72 qualitative KIIs were conducted with purposively sampled CHWs and policy/program stakeholders across the three countries working in community health systems–in managing, supporting, and providing direct services. Specifically, we apply a critical case sampling strategy that seeks particular information from varied perspectives related to the phenomenon of those embedded within community health during COVID-19 [25]. This comprised 25 CHWs and 13 stakeholders in Kenya, eight CHWs and 20 stakeholders in Bangladesh, and six CHWs and six stakeholders in Haiti. The CHWs include a subset of those surveyed quantitively and cover several subnational geographies in each country. All respondents played some role in the community health response to COVID-19 and sustaining routine service provision. Sample sizes were guided by thematic saturation around experience with COVID-19 –of which our participants had rich experience and in-depth information [26]. The KII guide probed open-endedly at relationships, experience within the COVID-19 response, and contextual circumstances that relate to trust between CHWs, clients, and other health structures (S1 Text). Qualitative interviews were audio recorded, transcribed and translated into English.

### Data analysis

Survey data analysis was conducted in Stata (Version 16, StataCorp, College Station, Texas). Measures employed are evident from the tables; measures related to CHWs’ positive and negative experiences during COVID-19 –including frequency and type of interaction–were newly developed by the research team given nascency of the pandemic. Trust was measured through an item assessing CHW perspectives of community trust in information provided by CHWs. We conducted all analyses separately by country, and chose not to statistically test differences in findings between countries given differences in sampling strategies and geographic coverage. For descriptive analyses we generated frequencies/means and measures of dispersion for each variable. We also constructed two regression models, to examine associations with positive and negative experiences of CHWs during COVID-19, The first model was for index of positive experiences (a count variable ranging from 0 to 5—see Table 2), for which we used Poisson regression and estimated Incident Rate Ratios (IRR). The second model was for negative experiences (No; Yes, a little; Yes, a lot), for which we used original logistic regression and estimated odds ratios. We estimated both the unadjusted and adjusted effects estimates for most socio-demographic and work characteristics from Table 1, as well as reported community trust in CHWs for COVID-19-related information (shown in Table 2).

**Table 1:** CHW socio-demographic and work characteristics.

	Kenya	Bangladesh	Haiti
	(n = 1,385)	(n = 370)	(n = 261)
*Community health worker characteristics*			
Age–mean (range)	44.7 (19–78) years	44.4 (19–58) years	46.3 (19–65) years
Female gender	68.3%	80.5%	(43.7%
Highest level of education completed			
Less than secondary	36.8%	23.2%	14.9%
Secondary	10.6%	75.1%	10.0%
More than secondary			
Rural (vs. urban/peri-urban)	74.4%	73.2%	85.8%
Lives in the community s/he works in	96.4%	68.7%	92.0%
Length of time working as a CHW			
<5 years	28.6%	4.1%	20.7%
5–10 years	40.7%	9.5%	52.5%
>10 years	30.7%	85.4%	26.8%
Continued routine work during COVID-19 pandemic ^a^			
No	2.7%	0.3%	5.1%
Yes, somewhat	38.7%	12.2%	70.6%
Yes, mostly	58.5%	87.5%	24.3%
Provides services related to COVID-19^b^	92.1%	37.8%	96.9%
Service provision modalities during COVID-19^c^			
Door-to-door/household visits	84.7%	97.6%	91.2%
Over phone calls (speaking)	47.0%	97.6%	33.7%
Digital communications (SMS, WhatsApp)	23.0%	3.8%	13.4%
Community meetings/health talks	39.1%	66.2%	85.4%
Provide services from a facility/clinic	39.7%	79.7%	32.2%

^a^ This measure combines responses to questions regarding the extent of continuing routine work in the first month after the COVID-19 pandemic began, and currently (in the month before survey)

^b^ This measure includes prevention, referral and/or reporting services

^c^ Of note, reported service provision modalities during the COVID-19 pandemic were similar to those pre-pandemic (also assessed on the surveys).

**Table 2:** Trusted CHWs and positive community interactions 6–8 months into the pandemic.

	KENYA	BANGLADESH	HAITI
	(n = 1,385)	(n = 370)	(n = 261)
Trust in CHWs as information source	Mean	Mean	Mean
	(SD)	(SD)	(SD)
On a scale of 1 to 10, how much do communities trust the information **you, as a CHW**, give them about COVID-19 (1 being the least and 10 being the most)? ^a^	7.92	7.84	5.65
	(4.05)	(1.22)	(2.34)
On a scale of 1 to 10, how much do communities trust the information they get from the **government/ Ministry of Health** about COVID-19? [via SMS, radio, newspapers, etc] ^a^	7.28	7.81	3.35
	(6.72)	(1.74)	(1.97)
**Positive interactions experienced as a CHW during COVID-19** *(spontaneous responses)*	**%**	**%**	**%**
Communities/individuals are relieved to see me as a CHW	62.7%	69.7%	88.5%
I feel I have been able to help keep my clients’ spirits up	62.5%	41.6%	64.8%
I feel I have been able to help my clients prevent COVID^a^	73.3%	28.6%	36.8%
I feel I have been able to help my clients get the healthcare they need during COVID	61.5%	37.0%	2.3%
Community members have expressed appreciation for CHWs as an important resource in these times	36.3%	13.0%	0%
**Total number of positive interactions experienced:**			
0	6.4%	0.3%	11.5%
1	13.6%	43.8%	23.8%
2	18.9%	31.9%	28.4%
3	20.9%	16.0%	34.1%
4	19.7%	7.6%	2.3%
5	20.4%	0.5%	0%

^a^Among CHWs reporting providing services related to COVID-19 (n = 1,276 in Kenya, 140 in Bangladesh, and 253 in Haiti)

Qualitative data analysis drew on a grounded theory principles and framework approach that involved inductive and deductive exploration of themes related to CHWs [27, 28]. Country and U.S.-based analysts familiarized themselves with the transcripts, recorded emergent themes and an inductively developed country aligned codebook. The codebook was finalized through deliberative reflection by multi-country team members relating themes to the overarching research objectives exploring CHW ability to work and experience during the COVID-9 pandemic (e.g. assessing routine and added responsibilities and support; and exploring the negative and positive experiences of CHWs interacting with communities and health facilities). One or two coder per country applied this codebook to all transcripts in QSR International’s NVivo 12 software (released in March 2020). This study focused on themes exploring CHWs recounting of their experiences interacting with communities and facilities, including the relationship with trust. We systematically reviewed relevant code reports related to positive and negative CHW experiences–including trust, hostility, challenges, and institutional support. Generating a matrix that charted emerging themes and illustrative quotes across the three countries enabled interpretation of the data. Finally, a constant comparative and iterative approach was used to synergistically interpret and integrate quantitative and qualitative findings [29].

## Results

### Respondent characteristics

Characteristics of survey respondents are shown in Table 1. Mean age of CHWs was about 45 years in each country. A majority of CHWs were women in Kenya (68%) and Bangladesh (81%); 44% were women in Haiti. Less than half of CHWs had completed at least a secondary education, in Kenya and Haiti, whereas three-fourths had completed more than a secondary education in Bangladesh. Nearly all CHWs in Kenya and Haiti lived in the community they work in, as did over two-thirds in Bangladesh. With regard to the work CHWs were engaged in during the COVID-19 pandemic, the vast majority reported (≥95%) continuing their routine work. In addition, in Kenya and Haiti, over 90% reported providing services related to COVID-19, as did 38% in Bangladesh. A high proportion (>80%) of CHWs in each country reported providing door-to-door/household visits during the pandemic. Finally, most also reported providing services via other modalities, including by phone, SMS/WhatsApp, community meetings, and at facilities.

### Trust as enabling and positive experience

Both the qualitative interviews and surveys showed that, across all three countries, prevailing pre-pandemic community trust in CHWs enabled CHWs to serve as integral stakeholders in disseminating information and providing services during the COVID-19 pandemic. Qualitatively, CHWs described their ability to parlay trust into action given their residence in the communities they serve, as well as community appreciation for their role as service providers, counselors, and respected community members.

*I try to be straight forward and honest with people in the community*, *I do not give them the run around*. *These people have placed their trust in me; we live in the same community and we work together*, *wherever they see me*, *they call me*. *I am always at their service… Even during the pandemic*, *whenever I come*, *they are always asking*: *“Did you bring any sanitizers*, *alcohol*, *this or that…”*. *They really appreciate me*, *and I appreciate them*, *and it is the way I am that makes it easy for people to adapt to me*. (CHW, Haiti)

*Even during this pandemic period they trust us*, *when you go to speak to them they listen which is good*. *When you go to other places [outside the community you work in] you might even be stoned and chased away [by communities] saying that there is no Covid-19 and that you are lying to them*. *But you see [here] that they sit and listen to us*. (CHW, Kenya)

Survey data (Table 2) showed relatively high levels of community trust in CHWs as sources of information about COVID-19—rated on average almost 8 on a scale from 1 to 10 in Kenya and Bangladesh, and 5.6 in Haiti. Such trust in CHWs was even higher than trust in information received from Ministries of Health (although only marginally so in Bangladesh). CHWs reported several positive interactions as a CHW during COVID-19 (noting these data were based on spontaneous response). A large majority in each country said that communities are relieved to see them as CHWs, and (in Kenya and Haiti) that they feel they’ve been able to keep their clients’ spirits up. Other prevalent responses in Kenya included feeling they’ve been able to help their clients prevent COVID-19 and get necessary care during the pandemic. In general, more positive experiences were noted by CHWs in Kenya than in Bangladesh or Haiti.

In bivariate analyses with the index of positive interactions as the outcome (Table 3), significant associations were found with reporting more positive experiences, for secondary education (vs. less than secondary, Kenya, p<0.01), continuing routine work during the pandemic (Bangladesh, p<0.05), and providing services related to COVID-19 (Kenya, p<0.001). In multivariate analyses, significant associations were found for older age (Bangladesh, p<0.01), secondary education (vs. less than secondary, Kenya, p<0.01), and providing services related to COVID-19 (Kenya, p<0.001).

**Table 3:** Results from Poisson regression model with index of positive experiences as the outcome.

Outcome: Count of positive experiences during COVID-19 (possible range: 0–5)	Kenya		Bangladesh		Haiti	
	(n = 1,385)		(n = 370)		(n = 261)	
	IRR	aIRR	IRR	aIRR	IRR	aIRR
Age	1.000	1.000	0.993	0.986**	0.953	0.947
Female (vs. male)	1.035	1.029	1.154	1.154	0.899	0.903
Highest level of education completed						
Less than secondary	(ref)	(ref)	(ref)	(ref)	(ref)	(ref)
Secondary	1.106**	1.112**	0.688	0.719	0.997	0.978
More than secondary	1.049	1.078	0.768	0.713	1.091	1.038
Rural (vs. urban/peri-urban)	1.025	1.005	1.106	1.093	0.924	0.899
Lives in the community s/he works in	1.026	0.738	1.038	1.019	0.856	0.855
Length of time working as a CHW						
<5 years	(ref)	(ref)	(ref)	(ref)	(ref)	(ref)
5–10 years	1.061	1.064	0.829	0.849	1.036	1.059
>10 years	1.050	1.060	1.012	1.280	0.991	1.058
Continued routine work during COVID-19 pandemic ^a^	1.025	1.012	1.357*	1.238	0.927	0.886
Provides services related to COVID-19	1.274***	1.266***	1.064	1.086	n/a^b^	n/a^b^
Trust in CHWs as information sources (scale of 1–10)	1.000	1.000	1.034	1.019	0.974	0.966

*p<0.05 **p<0.01 *p<0.001

IRR = Incidence Rate Ratio, aIRR = Incidence Rate Ratio, controlling for all other independent variables

^a^ Recoded as binary given low proportion responding no; binary categories are no/Yes, somewhat vs. Yes, mostly

^b^ Lack of variation in variable

Qualitatively, community trust contributed to CHWs’ sense of pride in their work especially around COVID prevention education activities. For example, across all three countries, some CHWs and program stakeholders attribute the low number of people with COVID-19 in their catchment area to CHWs’ success educating communities on avoiding transmission by engaging in protective behaviors like mask-wearing, handwashing, social distancing, and managing symptoms. Many CHWs modeled protective behaviors within their interactions with clients–e.g., “*I try to keep people at a distance because I did not want to catch the virus*”–and felt that persistence in reiterating messages promoted better understanding of COVID-19 risk.

*I am happy that when I go to the village*, *people listen to me*. *I am also glad to see that in my area there aren’t any positive COVID-19 cases reported and people have adhered to the rules of wearing masks and such so we don’t get cases* (CHW, Kenya)

*How do I feel*? *I am proud because if someone had died of the virus because I did not inform them*, *I did not educate them*, *I did not talk to them*, *I would feel awful*. *But given that we did the services the way they [government and non-government programs] asked us to do them*, *we followed through*, *makes me proud*. (CHW, Haiti)

*The direct impact is that [CHWs] have worked to build awareness among the people so that they can remain safe from the Corona*. *Maybe there is no percentage or survey data at my hand from which I can tell you how much people were aware of Corona from our workers*, *but people have followed the rules like wearing masks and staying at home as much as they could*. *This is a huge contribution of the health workers to prevent contamination in the first phase*. (Policy/program stakeholder, Bangladesh)

The protective effect of trust during the pandemic was reported over time by CHWs and program stakeholders alike in that CHWs felt their familiarity with clients opened up community access in ways unattainable by other external information sources and health providers.

*Personally*, *they have not barred me from getting into the houses…they are my community members*, *so they know me*. *There is nobody that doesn’t know me in my community unit*. *I am not a visitor*. *If you are a visitor*, *that is when you can have challenges*. (CHW, Kenya)

In Haiti, the emotional support and accompaniment to referral facilities that CHWs provided community members during the pandemic was critical to promote well-being. Moreover, CHWs described working to dispel myths, which they linked with helping prevent discrimination against those with the virus.

*We did not have any [hostility] in my neighborhood*, *especially with the way I talk with them*, *speaking with gentleness*, *they tend to listen to us… Wherever I go*, *once I talk*, *they are always ready to ask me questions*. *Whatever the issue*, *they feel comfortable enough to share them with me*. (CHW, Haiti)

*I wasn’t afraid because whatever preventative measures I had to take; I took them*. *We always find a way to reach people*, *help them protect themselves*, *instruct them not to shun the person who contracted the virus*. (CHW, Haiti)

### Negative CHW experiences: Navigating community fear and hostility

The positive, protective effects of community trust in CHWs during the pandemic was sometimes challenged by CHWs’ negative experiences while working with communities. Survey data (Table 4) showed that about one-third of CHWs experienced hostility or mistreatment from their communities because of their work as a CHW in the months preceding the survey. Among those who reported experiencing any form of hostility/mistreatment, most reported these happening “sometimes” rather than “often”. The most frequently cited type in Bangladesh and Haiti was being refused entry into people’s homes (>90%); almost two-thirds reported the same in Kenya. Another common type was clients ignoring CHWs’ advice/messages (over half reported this in Kenya and Bangladesh and one-quarter did so in Haiti). In Kenya, 59% reported being shouted at/spoken to harshly; 34% in Bangladesh and 17% in Haiti reported the same.

**Table 4:** Negative experiences in the community reported by CHWs 6–8 months into the pandemic.

		KENYA	BANGLADESH	HAITI
		(n = 1,385)	(n = 370)	(n = 261)
Hostility/mistreatment experienced by CHWs from community	Response categories			
Have you experienced any hostility or mistreatment because of your work as a CHW in the last 2–3 months (during COVID)?	No	69.2%	75.1%	64.0%
	Yes, a little	28.7%	23.8%	29.5%
	Yes, a lot	2.0%	1.1%	6.5%
*Which if any of the following types of hostility have you experienced* *because of your work as a CHW* *in the last 2–3 months (during COVID-19)*?		(n = 426)	(n = 92)	(n = 84)
My clients did not want to hear my advice/messages.	Sometimes	58.2%	52.2%	25.3%
	Often	12.0%	2.2%	1.1%
I was refused to enter clients’ homes	Sometimes	48.8%	87.0%	98.9%
	Often	10.1%	3.3%	1.1%
I was yelled at/spoken to rudely in the community	Sometimes	54.0%	32.6%	16.7%
	Often	5.4%	1.1%	0%
I was prevented from entering the community I normally work in	Sometimes	16.4%	23.9%	4.6%
	Often	2.4%	2.2%	1.2%
People in the community said hostile things about me behind my back/gossiped about me	Sometimes	31.5%	3.3%	20.2%
	Often	6.6%	1.1%	1.2%

In bivariate analyses with the index of negative experiences as the outcome (Table 5), significant associations were found with reporting fewer negative experiences, for older age (Kenya, p<0.01, Bangladesh, p<0.001), more than secondary education (vs. less than secondary, Kenya only, p<0.01), rural location (vs. urban, Kenya only p<0.001), 5–10 years working as a CHW (vs. <5 years, p<0.05), continuing routine work during the pandemic (Kenya and Haiti, both p<0.001), and trust in CHWs as information sources (Kenya and Bangladesh, p<0.05). In multivariate analyses, CHWs reporting fewer negative experiences was associated with older age (Kenya, p<0.01, Bangladesh, p<0.001), rural location (vs. urban, Kenya only p<0.001), continuing routine work during the pandemic (Kenya and Haiti, both p<0.001), providing services related to COVID-19 (Bangladesh, p<0.05), and trust in CHWs as information sources (Bangladesh, p<0.01).

**Table 5:** Results from ordinal logistic regression model with hostility/mistreatment as the outcome.

Outcome: Hostility/mistreatment during COVID-19 (No; Yes, a little; Yes, a lot)	Kenya		Bangladesh		Haiti	
	(n = 1,385)		(n = 370)		(n = 261)	
	OR	aOR	OR	aOR	OR	aOR
Age	0.985**	0.983**	0.955***	0.938**	0.980	0.954
Female (vs. male)	0.966	1.002	1.014	0.988	0.931	0.984
Highest level of education completed						
Less than secondary	(ref)	(ref)	(ref)	(ref)	(ref)	(ref)
Secondary	1.027	0.973	1.000	1.000	0.920	1.137
More than secondary	1.641**	1.260	1.000	1.000	0.791	0.633
Rural (vs. urban/peri-urban)	0.559***	0.657**	0.687	0.611	0.636	0.516
Lives in the community s/he works in	0.739	0.961	1.045	0.904	0.943	0.954
Length of time working as a CHW						
<5 years	(ref)	(ref)	(ref)	(ref)	(ref)	(ref)
5–10 years	0.747*	0.876	0.793	1.063	0.704	0.685
>10 years	0.933	1.123	0.492	1.268	0.853	0.777
Continued routine work during COVID-19 pandemic ^a^	0.555***	0.577***	1.241	1.453	0.363**	0.327**
Provides services related to COVID-19	1.128	1.492	0.615	0.489*	n/a^b^	n/a^b^
Trust in CHWs as information sources (scale of 1–10)	0.931*	0.969	0.866*	0.822**	1.067	1.020

*p<0.05 **p<0.01 *p<0.001

OR = Odds Ratio; aOR = adjusted Odds Ratio, controlling for all other independent variables

^a^ Recoded as binary given low proportion responding no; binary categories are no/Yes, somewhat vs. Yes, mostly

^b^ Lack of variation in variable

Qualitative data corroborated quantitative findings with respect to CHWs’ experience of hostility, and further showed that across countries, varied beliefs and fears dictate the types of hostility experienced by CHWs. Milder forms of hostility arise with fears of contracting COVID-19 from CHWs and manifest in negative client reaction (refused entry into one’s home), while the denial that COVID-19 exists leads to more moderate hostility (being shouted at), and/or avoidance of preventative behaviors (ignoring advice/messages). Community reactions to CHWs have varied implications for trust, where clients and CHWs make risk calculations (COVID-19 exposure versus service benefit), based on assumptions around COVID-19’s existence and availability of protective resources. For example, negative CHW experiences based on community reactions was described as lessening over time in Kenya, Bangladesh and Haiti, with some re-emergence following local surges in COVID cases.

*Before*, *there was this hospitality like giving a chair to sit*, *bringing snacks etc*. *But in the middle of the pandemic they tried to avoid us most of the time*, *we were uncomfortable too*. *But as time went by*, *through counseling the situation became normal again in June/July*. *Now they don’t mind when we visit house to house but the mocking about Covid-19 is still there…*. *///later segment///… When I and some volunteers went to the field the elderly people of the community used to talk like why we ‘come here every day*. *They don’t need to hear these every day*.*’ They used to mock us*. *Sometimes we felt really bad*. (CHW, Bangladesh)

While CHWs find frequent mildly hostile experiences expected and reasonable given fears of COVID-19 transmission, they also find that these encounters strain their cultivated trusting relationships with communities. The fear of CHWs bringing COVID-19 to communities was ubiquitous in all settings, especially in Kenya and Bangladesh. CHWs perceived that clients engaged in a kind of COVID-related risk-benefit calculation, with certain services such as family planning and antenatal care being more acceptable when weighed against perceived COVID transmission risk posed within a household visit, compared to child nutrition education which may not be worth the risk. This risk-benefit calculation was also affected by any tangible forms of assistance CHWs brought with them (e.g., masks, sanitizer, help linking to food aid).

*One time I went to a home and the gates were closed… I knocked and the owner came out*. *He was harsh and told me that everyone was asked to stay at home*. *‘You are the one who is spreading corona*.*’ I told him I was a CHV and I have information*, *and he said ‘not now*, *you go*.*’ He asked me if I had come with soap and I told him no…*.*People stop trusting you and things have become hard*, *the trust goes down*. (CHW, Kenya)

In Haiti, where a fragile sociopolitical environment prevails and already widespread poverty is further exacerbated during COVID-19, community members’ financial and material burden gave rise to hostility and resistance against CHWs more than COVID-19 per se. In Kenya, where CHWs lack formal salaries, their increased responsibility as resource brokers in the community is further challenged by COVID-19. CHWs in Haiti and Kenya expressed frustration with being unable to assist families financially or instrumentally to protect themselves from COVID-19 –feeling as if they are letting clients down and breaking community trust.

*When people were not accustomed to the pandemic*, *did not want to wear masks*, *and showed at the assembly posts*, *they felt humiliated [and thought by our wearing masks] that we were afraid of them*. *They [community members] would go*: *“Why don’t you buy masks and hand them out for free*? *You have money*, *you could do it*.*” I did not have money to go out and buy people masks*, *this was difficult for me*. (CHW, Haiti)

*You are forced to share the little you have with them [community members] since they can’t help you for free and they also don’t have the food*. *While on my side I have not been paid*, *I just moved out early to go and hustle*. *Even in that hustle there are some days where you won’t find anything so you just come home to the children with nothing*. (CHW, Kenya)

Outright hostility or aggression toward CHWs, including harsh refusal to comply with CHWs advice–though infrequent–was particularly difficult for CHWs to deal with and challenged established trust. Such cases commonly involved individuals denying COVID’s existence and refusing referral for testing or advanced care at the hospital. CHWs and program stakeholders perceived such hostility to arise from beliefs that COVID-19 is a government hoax (Kenya) or that it was brought to rural areas from the wealthier city-dwelling and global travelers (Haiti, and somewhat in Kenya), or from a general frustration with the government’s perceived inadequate response to COVID and related socioeconomic needs (Haiti and Kenya).

*In certain [urban] areas*, *they [those who denied COVID-19’s existence] gathered in a well-known space and burned two pails that the government had given to reinforce hygienic measures*. *By doing so they show outright denial of the virus and refusal to follow preventative measures… in the beginning*, *they [COVID-19 cases] were imported cases–from those with means*, *who could travel abroad*… *Now*, *the rural areas–for the farmers with no travel*, *you cannot tell them to wear masks… they tell you*, *‘we already have financial worries and now have to worry about this*?*’… The first time we met the farmers*, *there were many threats*, *we needed an escort*, *they even threw stones at us*. (Policy stakeholder, Haiti)

*Even if they refuse*, *you keep on talking to them until there is an agreement*. *Sometimes on the streets we might talk to a person but that person assumes that he is being lied to and that there is no Covid-19…But you talk to them slowly until they understand… That is how we have been trained*. (CHW, Kenya)

In Bangladesh, overt hostility towards CHWs was less commonly noted and manifested differently. It emerged in the disrespect COVID-positive community members showed toward CHWs, or in instances where lockdown measures led to social marking (e.g., red flag) and potential stigmatization of COVID-positive individuals.

*He was a policeman and Covid-19 positive*. *I asked him to keep distance and give his information list*. *Then he said the people like us [CHWs] don’t have any value to him*. *After that I called some people of the committee and complained to them about him*. *They said I should not get bothered about this*. (CHW, Bangladesh)

*But those [people] who were positive*, *the Upazila administration immediately locked the house and hoisted a red flag*. *The lock-down was ensured by the office staff*, *administrative people*, *and the police force*. (Policy/program stakeholder, Bangladesh)

In many instances of CHW-experienced hostility across all three countries, pre-existing trust and continued communication helped ease clients’ fears and mitigate the more problematic cases as the pandemic wore on. In contexts where COVID-19 cases have been quite rare, such as in Haiti compared to Bangladesh and Kenya, CHWs’ ability to negotiate prevention measures such as social distancing is particularly difficult.

*This person was mingling with others yet there were speculations this is a person infected with Corona*. *The CHW went there and the individual got mad questioning*, *‘how did the CHW know I’m infected*? *Why are you coming to my home*?*’ So the CHW explained… after that*, *they calmed down and said*, *‘It is true*, *I was diagnosed*…*’ The CHW educated the patient that it is not good to walk around because you’re risking your relatives and everyone in the home*. *After that the patient agreed to stay indoors*. *(*Policy/program stakeholder, Kenya)

*When things got to the community level*, *time takes care of everything*, *as such today*, *the situation is calmer today*. *Today*, *we are at the lowest phase*, *in the sense that provisions were centered on the fatal aspect of the virus*, *reality is something totally different*. *Now*, *it has become more difficult to convince people that the virus is in the country*. (Program stakeholder, Haiti)

### CHWs as intermediaries: Relationships with health facilities and systems

The pandemic exacerbated difficulties faced by CHWs in their intermediary role navigating their communication and work, representing both the community and the formal, government-led and/or NGO-supported health system. Quantitative data reveal generally positive relationships between CHWs and health facility-based providers, with some experienced hostility (Table 6). Over one-quarter (26%) of CHWs in Haiti reported experiencing hostility or mistreatment from facility-based providers (most of those ‘a little’ rather than ‘a lot’), whereas only 5% in Kenya, and 2% in Bangladesh did so. The most common types of negative experiences reported across countries were CHWs feeling their questions remained unanswered, and that they were shouted at/spoken to rudely, followed by their referrals being ignored not being allowed to accompany their clients to providers. Given the low reporting of negative experiences with facility-based providers, we did not conduct multivariate analyses for this outcome.

**Table 6:** Negative experiences with facility-based providers reported by CHWs 6–8 months into the pandemic.

	Response categories	KENYA	BANGLADESH	HAITI
		(n = 1,385)	(n = 370)	(n = 261)
Have you experienced any hostility or mistreatment by facility-based providers in your work as a CHW during the COVID-19 pandemic?	No			
	Yes, a little	95.3%	98.4%	73.6%
	Yes, a lot	4.5%	1.4%	22.2%
		0.2%	0.3%	4.2%
*Which if any of the following types of hostility/mistreatment have you experienced by facility-based providers during COVID-19*?		(n = 65)	(n = 6)	(n = 69)
My questions remained unanswered	Sometimes	46.2%	66.7%	40.6%
	Often	9.2%	0%	2.9%
I was yelled at/spoken to rudely	Sometimes	44.6%	33.3%	26.7%
	Often	6.2%	0%	0%
My referrals were ignored	Sometimes	32.3%	16.7%	22.7%
	Often	12.3%	0%	3.0%
I wasn’t allowed to accompany patients to facility for any service	Sometimes	12.3%	50.0%	10.2%
	Often	6.2%	0%	3.4%

Qualitatively, CHWs described intermittent interactions with facility-based providers during the pandemic. Facilities or health system stakeholders were often seen by CHWs as sources of COVID-19 information and supplies, though the latter were not always accessible.

*Besides family planning methods we’ve counseled people regarding these matters in the time of COVID-19*. *The office provided us flip charts and banners where every symptom of COVID-19 were mentioned*. *What precautions should we take and how we can be safe were also mentioned*. (CHW, Bangladesh)

Our data suggest that when a client receives poor treatment at health facilities or hospitals, it also reflects poorly on CHWs. In some cases, particularly in Bangladesh, we found communities rejecting CHWs because of their connection to the facility. In Haiti, CHWs described feeling a sense of humiliation when facilities did not honor referrals during the pandemic, and voiced complaints accordingly during interviews and in routine supervisory meetings. In other settings, hospital staff reportedly feared getting the virus from CHWs or clients seeking care. In all such cases, trust relationships between CHWs, facility-based providers, and clients seemed to be affected; at times hindering client trust of CHWs.

*They [clients] told them [CHWs] that as they were working in the hospital so they couldn’t go inside their houses*. *This is a huge obstacle for them to work*. (Policy/program stakeholder, Bangladesh)

*When you refer the person to a certain place*, *they [facility-based providers] tend to disregard the reference*, *the patient will come to you and say*: *“I don’t know why you sent me to that place*, *they did not treat me well”*. *At the monthly meetings*, *we are quite vocal about this*, *[that] at times*, *we feel humiliated when we refer the patients to the hospital*, *they treat them badly*. *It is as if we do the referrals just to do them*. *For example*, *when we send the patients with the referral form*, *the people there are supposed to read it to see what kind of ailments the person is suffering from and provide them with care*. *I mean they don’t even take the form from the person…we are always running into patients who complain about this*, *and we have brought up the subject with the nurses*, *plenty of times*. (CHW, Haiti)

“*Some of the staff were afraid to catch the virus*. *They [CHWs] referred people to the hospital but some of the staff were afraid even though they had gone through training*. *It may happen that the person did not like the reception they received*, *and when the person ran into a worker*, *they would air out their grievances*. (Program stakeholder, Haiti)

CHWs across countries described facing high expectations in their intermediary role from both health system stakeholders and community members, which they often felt unable to fully meet given their lack of decision-making authority and resources. In some cases where CHWs faced hostility or refusal by community members to comply with prevention or care-seeking advice, they asked for support from supervisors–often based in facility settings–through existing mechanisms. Some CHWs also reported receiving specific training for handling different types of people and negative reactions during COVID-19.

*They [CHWs] try to solve the problem of that person [patient]*, *and if they can’t solve the problem*, *then tell us [supervisors]*. *We give them [CHWs] advice*, *and if necessary*, *we send the patient to the doctor*. (Policy/program Stakeholder, Bangladesh)

*…you go from one household to another meeting with people with different attitudes and beliefs*. *And the good thing is that after the training we had for the ten days for Covid19*, *we learned very many techniques of communication and handling patients and bringing yourself to their situation and understanding*. (CHW, Kenya)

## Discussion

Our findings suggest that pre-pandemic client trust in CHWs serves as protective factor through the early phase of COVID-19 in overcoming negative CHW experiences and maintaining resilient community health systems in Kenya, Bangladesh and Haiti. Multivariate analyses revealed that the variables most consistently associated with CHWs reporting more positive experiences, and fewer negative experiences, were continuing their routine work during the pandemic, doing COVID-19-related work, and greater community trust. Qualitative findings showed that as CHWs continue interacting with communities to promote the government response at the frontline, their vulnerability increases with the public’s frustration with the pandemic and its associated restrictions. Across settings, mild to moderate hostility can escalate to overt aggression or retaliation toward a CHW, if compounded by context-specific environmental factors such as socioeconomic stress and political skepticism. In contrast, when CHWs are well-supported and adequately resourced, community trust in CHWs is strengthened. The bonds of trust within CHW-community relationships can withstand constraints and misinformation associated with perceived government response at the community level, particularly when CHW-facility/health system relationships reinforce CHWs in their intermediary role.

CHW experiences with communities and facilities appear generally positive and offer–vis-à-vis their trusted relationships–a resilient source of health services amidst the pandemic. This resonates with patterns of continued routine and added service provision seen in studies assessing health systems’ shocks in the face of disease outbreaks, including COVID-19 [30–32]. Our findings demonstrate CHWs sustaining their commitments across countries to protect individuals and communities from further pandemic-associated harms. Specifically, CHWs appear to mitigate stigmatization of COVID-19-positive individuals and diffuse community tensions in the face of stressed resource environments. The shifting information, governmental guidance, and supply deficiency exacerbation due to COVID-19 –stockouts of essential medicines and PPE–in our study parallel the challenges faced by CHWs and key stakeholders in the Ebola crisis in Guinea, Liberia, and Sierra Leone [33]. During the Ebola outbreak, shifting guidance led to widespread misinformation and depreciated community mistrust of health care workers. While our study documented some reduction in trust, the socially motivated community health stakeholders saw themselves as highly valued and respected resources during the COVID-19 pandemic. This exemplifies the promise of pre-pandemic trusted CHW-community relationships achieved through interpersonal communication and health information sharing [2].

Interestingly, our multivariate findings suggest that sustained CHW presence providing services in the community, including around COVID-19 speak to the protective associations with trust. There were few significant sociodemographic and contextual associations with positive or negative experience, unlike trust and sustained interactions with communities, for which significant findings were quite consistent. This suggests that CHWs may share perceptions of trust and related community factors (e.g. routine service provision and COVID-19-pecific work) across the three countries. Moreover, our findings around the levels, nature, and quality of positive and negative CHW-community interactions as closely related to the support available to CHWs to overcome challenges in their work resonates with CHW-focused pandemic studies elsewhere [34].

It is important to consider how pandemic trends, the stringency of government response across the three countries, and other context-specific sociopolitical factors between March and December 2020 may have affected our assessment of community and facility trust in CHWs. As noted previously, both the COVID-19 burden and stringency of lock-downs was highest in Bangladesh, moderate in Kenya, and low in Haiti [20–22]. During this time frame, in Haiti, multiple strikes and riots against the government as well as kidnappings for ransom raged across the country, spreading beyond the capital of Port-au-Prince. These sociopolitical factors alongside perpetual gang violence in the Artibonite department set a tone of general insecurity but also skepticism of government response to anything, including COVID-19. These factors in Haiti demonstrably show repercussions for diminished trust in the government, and to a lesser extent for trust in CHWs–compared to Kenya and Bangladesh. As the pandemic wears on, our data across all three countries showed that complacence and skepticism around shifting restrictions continue to affect community-CHW relationships. Further investigation about trust dynamics within CHW-facility-based provider relationships is needed and remains under-explored within our study.

Our study’s findings with respect to trust relationships as manifest in positive and negative CHW experiences during the COVID-19 pandemic point to several implications for programs and policy. First, CHW training ought to incorporate conflict resolution techniques at the community level, among other disease outbreak management strategies, such as adapting existing emergency preparedness modules within CHW curricula, formalization of CHWs generally and as a reserve health corps cadre, and early integration into government response [7, 33, 35–37]. Particularly in countries where CHWs have formalized support systems; building emergency preparedness skills in advance and providing ongoing training updates throughout an outbreak can increase their efficacies as community educators and mobilizers of protective behaviors. In the case of COVID-19, adaptive trainings could hone CHWs educational role towards promoting vaccine uptake. Our findings align with global perspectives advocating for CHW as key assets and potential leaders within community-based health systems to educate about and in some countries (where permissible) administer vaccines [38]. Second, leveraging CHWs’ intermediary role within community norms-shifting interventions can help destigmatize COVID-19 more broadly. Prior research on community interventions with those living with HIV and mental illness, highlights the relevance of CHWs’ intermediary roles in reducing disease-associated stigma though awareness promotion and socio-cultural advocacy within heath systems [39, 40]. Third, policymakers need to ensure governments’ dynamic and ongoing response to COVID-19. This includes mitigating the undue burden of PPE gaps in the community as points of tension for CHWs. It also involves financially protecting–via hazard pay or health insurance–CHWs for taking on added responsibility and health risks.

While this study was quick to respond to early pandemic experiences of community health stakeholders—including CHWs, there are limitations to its scope. Our data is subject to recall bias given we interviewed all participants at one point in time and retrospectively of their experience pre-and-during the first six to eight months of the pandemic. Our study is only representative of CHWs who have routine access to mobile phones, which precludes inclusion of more vulnerable and materially challenged CHWs. Given the dynamic nature of the COVID-19, future studies merit longitudinal analyses that were beyond the resource envelope for our study. The remote nature of data collection precluded our interviewing community members or clients themselves; doing so would have enabled us to gain a more direct perspective of trust in CHWs, broader government COVID-19, and longitudinally assess trust metrics over time as shifted into varying states of crises. Finally, given the cross-sectional and self-reported nature of the survey data, findings from regression analyses regarding associations with positive and negative experiences, cannot be interested as necessarily causal in nature,

## Conclusion

While COVID-19 pandemic and associate-government responses shocked and exacerbated existing health system challenges facing community health stakeholders, including CHWs, the bonds of trust between CHWs, communities, and facility-based providers were affected by positive and negative reactions in Bangladesh, Haiti, and Kenya. Community members’ calculus of feared transmission against protective assets provided by CHWs manifest at the CHWs level as calculus of risk of working v. government protections and support (vis-à-vis facility providers). Existing pre-pandemic trusting relationships withstood the early phase of COVID-19 –particularly through CHWs’ commitment to educating communities and mitigating misinformation, though longitudinal research is warranted to assess these dynamics in the same populations over time. The protective potential of trust in CHWs, as well as their change agent value in communities can be pragmatically supported and channeled into COVID-19 programming, including vaccine promotion.

## Supporting information

S1 Checklist Inclusivity in global research. (DOCX)

S1 Text Key informant interview guides. (PDF)
