# Supplementary material for: Confirming—and testing—bonds of trust: A mixed methods study exploring community health workers’ experiences during the COVID-19 pandemic in Bangladesh, Haiti and Kenya
Source: PLOS Glob Public Health. 2022 Oct 7;2(10):e0000595. doi: 10.1371/journal.pgph.0000595 (PMC10021319; doi:10.1371/journal.pgph.0000595)
Supplement: S1 Text — (PDF) [file pgph.0000595.s002.pdf]

POPULATION COUNCIL
Ideas. Evidence. Impact.

Frontline Health
Harmonizing Metrics. Advancing Evidence. Accelerating Policy.

S1_Text. Key Informant Interview Guides

Key Informant Interview Guide for Policy and Program Stakeholders

1. Tell us about the COVID-19 stage the country is in currently and how has changed over the past couple months.

2. How has COVID-19 affected the implementation of community health in this country?
   a. What are the current strategies being used? (Probe: guidelines around social distancing, behaviors at the community level, and for health workers working to prevent and treat COVID-19)?
   b. Tell us about any sub-national responses that may exist?
   c. How have governmental and non-governmental actors responded?
   d. Describe any coordination efforts between policy and program stakeholders (government, non-government, international agencies, donors) in the COVID-19 response? Any particularly related to community health?

3. Tell me about how you typically work with/ support community health systems and community health workers (CHWs)?
   a. How has that changed since COVID-19 started?
   b. Is there new guidance or policies around CHWs work? Please explain.
   c. Tell us how CHWs' scope of work has changed or expanded. (Probe: COVID education/health promotion, WASH activities, streamlining referrals, contact tracing, data reporting.)
   d. How are they working to mitigate the negative effects of COVID-19 on people's health formally and informally/spontaneously?
   e. How are they working with health systems/facilities?

4. What do you think are some of the challenges CHWs face in their work during COVID-19?
   a. How have these changed since the COVID-19 first started and now?
   b. What are some of the challenges to home-based or community-based care?
   c. Are CHWs facing any hostility or mistreatment by the communities they are trying to serve out of fear of catching the disease? Please explain (what types, give examples).
   d. Are CHWs facing any challenges in interacting/communicate with their supervisors and other facility-based providers? Give examples of what types of challenges.

5. How can policy and program stakeholders work together to support community health systems in the context of COVID-19 pandemic?

6. Please tell us about any innovative strategies being used in your country to help with the challenge of misinformation around COVID-19 affecting people's prevention and care-seeking behaviors? (Probe: call centers, TV, radio, other?)

Key Informant Interview Guide for the CHVs in Bangladesh, Haiti, and Kenya

1. Tell us about your daily experiences and responsibilities as a CHV during the COVID-19 Pandemic? **Probe for: how has that changed from the normal before COVID?**

2. Thinking back to how you provided care to communities over the past months during the COVID-19 period…
    a. *What routine health services have been you been able to provide in the community and how did you provide these services (e.g. education and primary health care, FP, MNCH, WASH, mental health)*
    b. *What type of health services were disrupted and why?*
    c. *We know you have been providing a lot of educational services around COVID-19 prevention and management in home/community, however you have trouble in reporting suspected positive cases and making referrals to advanced care. Please tell us more about this difficulty in reporting and referrals?*

Topic I: Understanding experiences of CHV while providing community and home-based services

3. What are some of the positive experiences you experienced while providing community and home-based care?
    a. Tell us about a time you felt good about the care you provided?
    b. What makes you feel respected by the community as a CHV during the pandemic? (give an example of when you felt respected, probe: any other?)

4. Tell us about any challenges you faced while providing community level services?
    a. *Was there a time you failed to provide services? Why were you unable to do so?*
    b. *Did you face any hostility or feel humiliated/disrespected by the community while providing services because of COVID-19 fears? Please explain (**Probe**: what types refused entry to the homes, spoken to rudely, refused community entry, gossiped any other?; how did this happen, can you give examples).*
    c. *Describe any disagreements with community members Points where communities refused entry to their homes or expressed disinterest in your support? How did you manage that?*
    d. *Were there any other challenging experiences while interacting with community members? Please give examples.*
    e. *How have these challenges changed since the COVID-19 first started and now?*

5. What strategies were you able to employ to ensure that you provided the needed health services? (***Probe for coping mechanisms, necessary social support)***

Topic II: Understanding social and health systems effects of COVID-19 pandemic on CHVs

6. Since you are linked to a facility, how would you describe your experiences with facilities that are you attached to during this time of COVID-19?
    a. *Have you faced any challenges interacting/communicating with facility-based providers? Give examples of challenges.*
    b. *Tell us about any form of hostility from facility-based providers and staff you face? (**probe** inability to access supplies, referrals refused services any other?)*
    c. *Are there any experiences of any form of disconnect with facility during the period?*
    d. *What support did you receive from the facility? (**Probe** for support provided by local facilities, e.g. PPE)*
    e. *What are other forms of disruptions did you experience?*

7. Now let us talk about how you are trained and receive information on COVID-19?
    a) *What information did you receive that helped you to navigate the pandemic and how did you use it?*
    b) *What areas of training did you undergo: Continuity of services, home-base care, COVID-19 Prevention and management? Other?*
    c) *How did you apply what you learned? What additional training/information would be helpful moving forward?*
    d) *How would you want to be informed as the panic evolves to enable you continue with the activities at community level*? (face to face, phone, Whats App, other?)

Topic III: Recommendation for engaging CHV during pandemics

8. What would you describe as meaningful participation of CHV to support such pandemic? **Probe** for:
    a) Type of engagement and platforms for such engagement
    b) mechanism/approaches/strategies options for engaging CHV
    c) Areas of engagement that require improvement

9. What are the barriers to working with facilities and communities during the pandemic?
    Probes: *lack of clear guidance in engaging CHVs, Perceptions of role of CHVs doing pandemic, environmental, any other concerns?*

Thank you very much for your time.
